# CXCR4-A Prognostic and Clinicopathological Biomarker for Pancreatic Ductal Adenocarcinoma: A Meta-Analysis

**DOI:** 10.1371/journal.pone.0130192

**Published:** 2015-06-19

**Authors:** Andreas Krieg, Jasmin C. Riemer, Leila A. Telan, Helmut E. Gabbert, Wolfram T. Knoefel

**Affiliations:** 1 Department of Surgery (A), Heinrich-Heine-University and University Hospital Duesseldorf, Duesseldorf, Germany; 2 Institute of Pathology, Heinrich-Heine-University and University Hospital Duesseldorf, Duesseldorf, Germany; University of Nebraska Medical Center, UNITED STATES

## Abstract

Adenocarcinomas of the pancreatic duct (PDAC) are characteristically aggressive tumors that are extremely challenging to treat as curative surgical resection, the definitive treatment, is seldom possible. Regretably, most patients are diagnosed with metastatic disease at the time of initial presentation. In addition, current chemotherapeutic concepts that are used for advanced disease stages show frustrating results. Thus, there is an urgent need to identify novel therapeutic molecular targets that are associated with PDAC disease. Recently, the chemokine receptor CXCR4 has been demonstrated to be highly expressed in metastatic PDAC. However, the results of the published data on CXCR4 and its association with clinicopathological variables and prognosis in PDAC seem to be heterogeneous. Consequently, to clarify the relevance of CXCR4 as a biomarker in PDAC we performed a comprehensive literature search by using PubMed and Web of Science databases to identify articles that focused on the expression of CXCR4 in PDAC by using immunohistochemistry. Subsequently, data from nine relevant studies, encompassing 1183 patients were extracted, qualitatively assessed, and entered into a meta-analysis. By using a random effects model, the pooled hazard ratio of the seven studies that reported on patients overall survival revealed a correlation between expression of CXCR4 and poor prognosis (HR 1.49; 95% CI: 1.04-2.14; *P* = 0.03; I^2^ = 74%). Although heterogeneity became evident, subgroup analyses confirmed the prognostic value of CXCR4 in PDAC, especially in high-quality studies that performed multivariate analysis. In addition, meta-analysis revealed a strong association of CXCR4 expression with the UICC stage (OR: 3.40; 95% CI: 1.67-6.92; *P* = 0.0007; I^2^ = 0%) and metastatic disease (N-status: OR: 2.55; 95% CI: 1.56-4.15; *P* = 0.0002; I^2^ = 26%; recurrence to the liver: OR: 2.80; 95% CI: 1.48-5.29; *P* = 0.001; I^2^ = 0%). Taken together, our meta-analysis suggests that CXCR4 represents a useful prognostic biomarker in PDAC and might therefore be evaluated as a potential therapeutic target in the treatment of metastatic cancer disease of the pancreas.

## Introduction

According to the SEER database, in the United States the incidence of pancreatic cancer was 12.3/100.000 in 2011 with an estimated 5-year relative survival rate of 6.7% [1]. Thus, pancreatic cancer represents 2.8% of all new cancer cases and is the twelfth leading cause of cancer related deaths. From the histopathological aspect, most of these tumors are pancreatic ductal adenocarcinomas (PDAC). To date, the only curative treatment is surgical resection. However, due to the biological aggressiveness only 10–15% of the patients with PDAC are initially diagnosed at a stage at which surgical resection can potentially be curative [2]. Moreover, for metastatic PDAC administration of gemcitabine remains the first-line therapy among the chemotherapeutic agents [3]. Although clinical trials in which combinational regimes such as FOLFIRINOX improved patients survival, a common problem is still the toxic side effects [4]. Importantly, this underlines the urgent need to identify novel therapies that selectively antagonize molecular targets, not only to improve patients’ survival but also to minimize the adverse effects of treatment. A first step toward the development of such targeted therapies is based on the identification of a druggable molecule by profiling tumors for alterations in expression levels of proteins that might be associated with tumor progression and poor survival [5].

In this context, the C-X-C chemokine receptor type 4 (CXCR4) has attracted considerable attention since its expression has been described in various gastrointestinal malignancies [6–8]. CXCR4 consists of 352-amino acids and interacts with the stromal cell-derived factor 1 (SDF-1), also known as CXCL12, by binding selectively [9, 10]. Under physiological conditions, CXCR4 plays a crucial role during organogenesis and regeneration [11]. However, in pancreatic cancer the CXCL12/CXCR4 axis has been functionally implicated in tumor progression by initiating tumor cell migration, invasion, angiogenesis, and putatively inducing metastasis [12–14]. Accordingly, the chemoattracting effect of CXCL12 on CXCR4+ pancreatic cancer cells might reflect a major cause for the formation of metastases directly where CXCL12 is highly expressed such as in lymph nodes, in the liver, lungs, and bone marrow [15].

Furthermore, previous studies on PDAC tissue specimen suggested that CXCR4 expression might represent a valuable biomarker as evidence for an association between CXCR4 expression and metastatic disease as well as patients’ survival was found [16, 17]. However, these results still seem to be controversial. Consequently, we initiated a comprehensive review of the literature and conducted a meta-analysis to assess the suitability of CXCR4 expression as prognostic and clinicopathological relevant biomarker in PDAC.

## Materials and Methods

### Literature search

After reading the current literature that focused on the expression of CXCR4 in PDAC, our aim was to use meta-analyses to elucidate the question of whether CXCR4, when detected by immunohistochemistry, can serve as a valuable prognostic and clinicopathological biomarker in PDAC. In addition, before the review process was conducted, the inclusion and exclusion criteria were defined as outlined in the paragraph “selection criteria”. To identify articles that investigated the expression of CXCR4 in PDAC, a comprehensive literature search was performed by screening PubMed and Web of Science databases on November 3, 2014. In order to find particularly those articles that investigated the protein expression of CXCR4 in PDAC, keywords and text words were used as follows: **(1)** pancrea* OR PDA and **(2)** cancer OR carcinoma OR neoplasm OR malignancy OR adenocarcinoma, and **(3)** CXCR4. In addition, review articles and textbooks that are relevant in this field as well as the references of the selected articles were reviewed for eligible literature.

### Selection Criteria

Study selection was performed by two independent investigators (A.K. and J.C.R.) during a multi-step process. First, only those studies that focused on a potential relationship between CXCR4 expression and survival data or clinicopathological factors were defined as eligible. Thus, all abstracts were intensively screened by the two investigators independently to ensure that only articles that met exactly these defined criteria were extracted. After a consensus process, full texts of potentially interesting abstracts were read by both investigators to assess independently the relevance of these articles for meta-analysis according to the following pre-defined selection criteria: **(1)** CXCR4 expression patterns were detected by immunohistochemistry in pancreatic cancer tissue specimen; **(2)** an association of CXCR4 protein levels with clinicopathological variables or survival data was statistically analyzed; **(3)** articles had to be written as full text paper in English; **(4)** either the hazard ratio (HR) for overall survival could be directly extracted from the article or was presented in a way that made an estimation possible; **(5)** clinicopathological variables were presented in a format that allowed us to estimate the odds ratios (OR); **(6)** in case of dual publication, the most detailed and informative study was selected to be included in the meta-analysis; **(7)** unpublished literature, conference proceedings, dissertations as well as trial registries were excluded. A final consensus meeting was arranged to discuss disagreements and to finally select those articles that both investigators assessed to be eligible.

### Data extraction

Study characteristics such as first author’s name, publication year, country of origin, number of patients as well as patients’ sex and age, histopathological characteristics, use of neo- or adjuvant therapy, source/clone of antibody with cut-off value and statistical method for survival analysis were extracted and recorded in separate databases by two independent extractors (A.K. and J.C.R.). In a consensus meeting, both investigators compared their databases and combined the results together to yield a final database.

### Quality assessment

The scientific quality of the included studies was rigorously and independently scored by two investigators (A.K. and J. C. R.) according to the quality scale for biological prognostic factors established by the European Lung Cancer Working Party (ELCWP) with slight modifications [18]. For each category, scientific design, laboratory methodology, and generalizability, a total of ten points and for the results analysis a maximum of eight points could be achieved. A theoretical total quality score of 38 could be reached. Finally, the quality score was presented as the percentage of the global score that was maximal achievable (0 to 100%) with higher values reflecting a higher scientific and methodological study quality. However, the ELCWP score category “results analysis” evaluates only articles that present survival analyses. In this case, the theoretical total quality score was defined as 30 and the percentage of the achievable global score was adjusted accordingly. The quality scoring from each investigator was compared for each study and if a discrepancy occurred a consensus score was agreed upon.

### Statistical analysis

The OR was calculated by using the Mantel-Haenszel method to analyze the relation between CXCR4 expression and clinicopathological parameters. An OR greater than 1 indicated that CXCR4 expression was more likely related with advanced UICC stage and depth of invasion, poor grade of differentiation, presence of lymph node or distant metastasis, and male sex. Therefore, expression of CXCR4 was compared between T1/T2 and T3/T4 tumors, UICC stages I/II and III/IV, or between well or moderately differentiated tumors and poorly differentiated tumors. For each comparison, the number of samples exhibiting positive expression of CXCR4 was set in relation to the sample size within each group.

When studies reported the HR with 95% confidence interval (CI), these data were pooled directly in the meta-analysis. However, for studies representing only Kaplan-Meier curves an indirect estimation for HRs and CIs became unavoidable as recently described [19, 20]. Therefore, survival curves were read and converted into raw data by using the software Engauge Digitizer version 4.1 (http://digitizer.sourceforge.net/). Afterwards, raw data were entered into a GraphPad Prism survival spreadsheet (GraphPad Software, Inc, La Jolla, CA, USA) which produced Kaplan-Meier curves for comparison with the published curves and HRs with 95% CIs. However, we had to assume a constant number of censored cases during follow-up. To confirm our data, HRs and 95% CIs were also calculated by the Tierny method [21]. Generic inverse-variance weighting and random-effect modelling was used to pool HRs. A HR greater than 1 indicated an association between CXCR4 expression and poor prognosis in patients with PDAC.

Cochrane’s Q test (Chi-squared test; Chi^2^) and inconsistency (I^2^) were calculated to analyze the statistical heterogeneity. [22, 23] Because we included studies with potentially unequal characteristics (i.e. different patient pools, protocols, follow-up strategies) we had to assume heterogenous study results. Thus, a random-effects model (DerSimonian and Laird) was used to construct the pooled ORs and HRs with 95% CI [24]. Subgroup and one-way sensitivity analyses were performed to test the stability of the meta-analysis. Meta-analysis including the preparation of graphs was completed using the Review Manager 5.0 software (http://ims.cochrane.org/revman). Funnel plots were prepared to graphically assess publication bias. Differences in quality scores between distinct subgroups were statistically evaluated by non-parametric tests in which a *P*-value <0.05 was considered to indicate statistical significance.

## Results

### Study selection and characteristics

**Fig 1:**
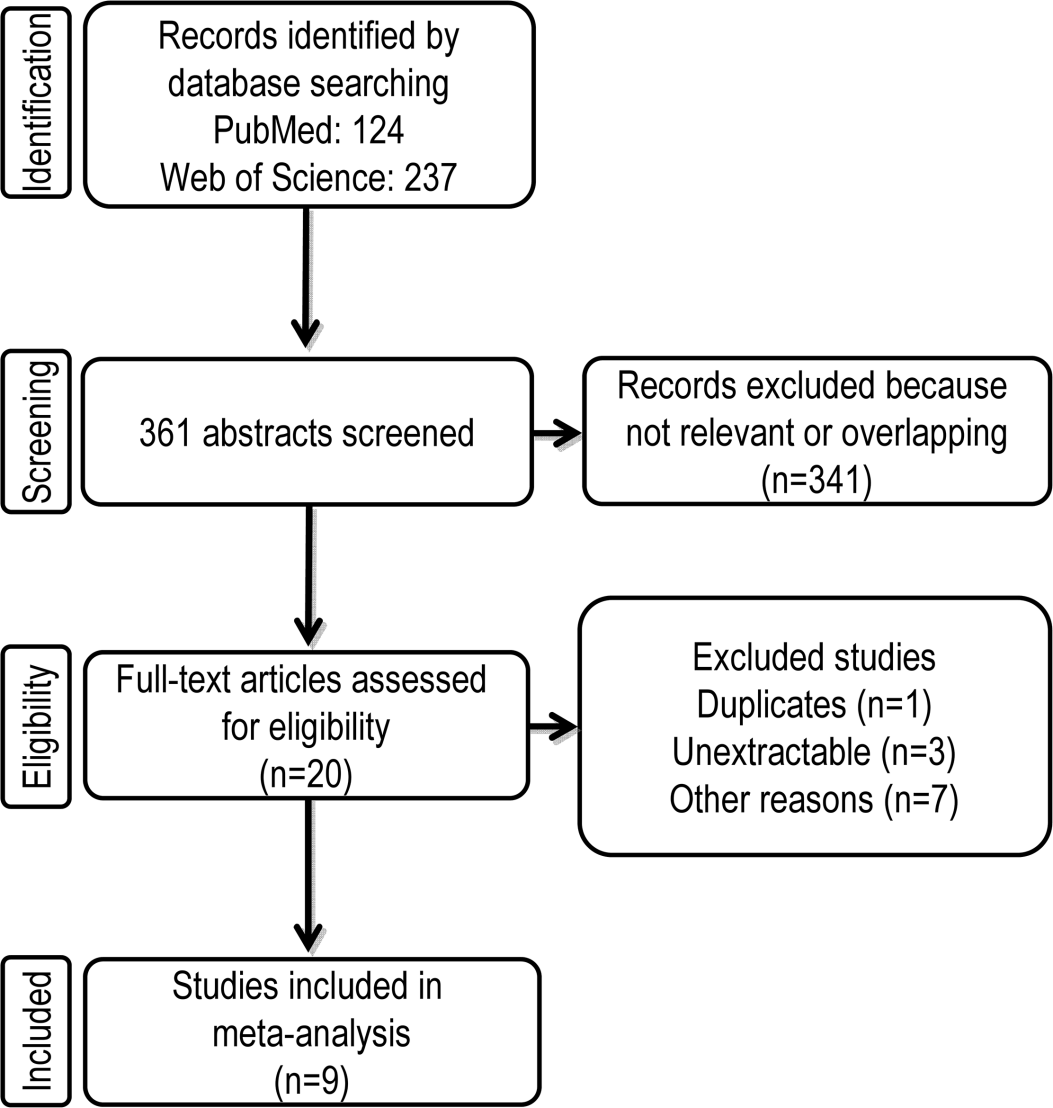
Flow chart summarizing the process of literature search and study selection.

Using our pre-defined keywords for the electronic database search, we retrieved 124 and 237 hits in the PubMed and Web of Science databases, respectively **(Fig 1)**. After carefully reading these 361 abstracts we found 20 studies that focused on the expression of CXCR4 in pancreatic cancer. Therefore, these potentially interesting articles were entered into the next step of full-text review. Our full-text review identified 11 articles that we excluded because of dual publication (n = 1) [25], non-extractable data (n = 3) [26–29], CXCR4 expression having been analyzed by techniques other than immunohistochemistry, [30–32] or because the CXCR4 expression was examined only in CD133-subpopulations [33]. Articles were also excluded when only data about perineural invasion [34] or distant recurrence [35] were extractable. For the latter two were excluded because there was only one included study investigating perineural invasion and a second study providing data about distant recurrence during follow up [16, 36]. Finally, nine studies that were published between 2000 and 2013 were eligible for our meta-analysis that aimed to elucidate the impact of CXCR4 as a prognostic and clinicopathological biomarker in pancreatic cancer [6, 16, 17, 36–41].

To briefly summarize the characteristics of included studies, important data were extracted by both investigators and synergized in a final table (**Table 1)**. Accordingly, five of the included studies were conducted in Asian countries, the remaining four studies in two different European countries. Because Gebauer and colleagues [6] excluded five patients and Bachet et al. [36] defined the whole population for multivariate analysis with 428 patients, the simple pooling of these data allowed us to include a total of 1183 patients (median: 97; range: 30 to 428) with PDAC in our meta-analysis. Four studies enrolled patients with PDAC of all UICC I-IV stages and two studies included only PDACs of UICC stages I and II. However, there were three studies that gave no information regarding the included UICC stages. Although most of the studies included patients treated by curative resection, there was one study that did not precisely report on the therapeutic intent of surgery, [38] and another study that included patients who received palliative surgery [40]. In addition, the study by Wehler and colleagues [41] included 13 patients with metastases who underwent endosonographic biopsy. However, only five studies provided sufficient information that indicated the exclusion of patients that were treated by neoadjuvant therapeutic regimes such as chemotherapy or radiotherapy [6, 17, 36, 37, 39]. In addition, five studies reported the number of patients who received adjuvant chemotherapy or radiochemotherapy [6, 16, 17, 36, 39]. Whereas the studies by Liao [16] and Gebauer [6] did not specify these adjuvant therapeutic concepts, patients in the studies published by Kure [39] or Bachet [36] received a gemcitabine monotherapy, gemcitabine in combination with other chemotherapeutic agents or other chemotherapeutic agents such as uracil, tegafur or fluorouracil. Out of the 71 patients that were included by Marechal et al. [17], 30 patients received adjuvant radiochemotherapy and 12 patients a gemcitabine monotherapy. However, in 2 studies that identified CXCR4 as a prognostic biomarker adjuvant chemotherapy did not affect overall, disease free or liver recurrence-free survival [17, 39]. In contrast, Bachet and colleagues identified expression of CXCR4 and chemotherapy as independent prognostic factors [36]. Importantly, in their subgroup analysis of patients who did not receive any adjuvant treatment, multivariate analysis identified high CXCR4 expression still to be associated with a poor overall survival [36]. Moreover, seven studies presented survival data, four of them provided data from multivariate analysis and three studies generated Kaplan-Meier curves that were used to extract survival data. In addition, one of these studies focused only on the prognostic value of CXCR4 in PDAC without comparing CXCR4 expression levels with clinicopathological variables [39]. Follow up frequency and examinations were reported only in two studies [16, 36]. In these studies, follow up was performed every 2–3 months including a clinical examination, laboratory tests with CA19-9 and abdominal ultrasound or Computed tomography scan. The median follow up was extractable from 4 studies and varied between 16.2 and 52 months **(Table 1)**. In all studies CXCR4 was detected by immunohistochemistry, which was performed on tissue microarray (TMA) slides in three studies [6, 17, 36]. Five studies clearly defined the antibody clone that was used for immunohistochemical staining [6, 16, 38, 39, 41]. The remaining four studies specified at least the antibody provider [17, 36, 37, 40]. Thereby, all studies but one revealed a predominately cytoplasmic expression pattern of CXCR4 **(Table 1)**. Only the study of Koshiba and colleagues found a positive staining of CXCR4 in the cytoplasm and/or cell membrane [38].

**Table 1:** Clinical and methodological characteristics of included studies.

First Author	PMID	Year	Country	Cases	Stage	Extracted Variables	Adjuvant therapy No. of patients (%)	Follow up Frequency (Months)	Follow up (Months) Median (Range)	CXCR4Localization	Cutt off value	HR Estimate	HR	95% CI
Koshiba	10999740	2000	Japan	52	I-IV	T,N,M,U	NS	NS	NS	cytoplasmic/ cell membrane	moderate	NA	NA	NA
Wehler	17089032	2006	Germany	103	I-IV	A,S,D,T,N, M,U	NS	NS	NS	cytoplasmic	strong	Sur. Curve (UV)	1.19	0.74–1.92
Maréchal	19352387	2009	Belgium	71	NS	A,S,D,T,N, M*,Le,Ve	42 (59)	NS	NS	cytoplasmic	EI score>3	MV	2.54	1.27–5.10
Cui	20462600	2011	China	30	I-IV	D,N,U	NS	NS	NS	cytoplasmic	upper quartile	NA	NA	NA
Gebauer	21520098	2011	Germany	254^§^	NS	A,S,D,T,N,M	230 (92)	NS	NS	cytoplasmic	5%	Sur. Curve (UV)	1.03	0.70–1.50
Kure	22824809	2012	Japan	105	NS	NA	59 (56)	NS	17.2 (0.4–153.1)	cytoplasmic	median (% of positive cells)	Sur. Curve (UV)	0.76	0.48–1.18
Liao	23238349	2012	Taiwan	97	I-II	A,S,D,T,N, M*,U,Lvi,Pn	17 (18)	3	26.3^Ф^	cytoplasmic	>20%	MV	1.78	1.02–3.09
Bachet	22377565	2012	Belgium	471^#^	I-II	M^$^	252 (59)	2–3	54 (1.1–143.7)	cytoplasmic	scale>2	MV	1.94	1.50–2.51
Wang	24023356	2013	China	48	I-IV	A,S,D,N,U	NS	NS	16.2 (NS)	cytoplasmic	NA	MV	4.88	1.38–17.24

Abbreviation: A, age; D, differentiation; Lvi, lymphovascular invasion; Le, lymphatic embolus; M, distant metastasis; N, lymph node metastasis; Pn, perineural invasion; S, sex; T, depth of invasion; U, UICC stage; Ve, vascular embolus; NS, not specified

* liver metastasis specified as recurrence

^$^, distant recurrence without organ specification

^#^ 428 patients in multivariate analysis

^§^ 249 patients evaluable

UV, univariate; MV, multivariate

^Ф^, only for 17 patients without recurrence or dead.

### Study Quality

The quality of each study was rigorously estimated in the categories study design, laboratory methodology, generalizability, and results analysis as described in materials and methods. Finally, a global quality score expressed the percentage of the total score that was theoretically achievable **(Table 2)**. Thus, the nine included studies reached a mean global quality score of 63.1% (range 53.3 to 86.8%). Next, we compared the categorical quality scores as well as the global scores between the studies in relation to the type of survival analysis (univariate *vs*. multivariate) or the country in which the study was initiated (Asian *vs*. European). In summary, statistical difference in the quality was not apparent in these comparative analyses. Because we included only two studies the aim of which was not to analyze the prognostic value of CXCR4, we refrained from comparing studies with and without survival data for statistical differences.

**Table 2:** Study quality assessment according to the ELCWP Scale.

		No. of studies	Design	Laboratory methodology	Generalizability	Results analysis	Global Score (%)
All Studies*		9	6.4	7.2	6.1	5.4	63.1
Survival Data		7	6.9	7.3	6.6	5.4	68.8
No Survival Data		2	5	7	4.5	NA	55
	P-value		^#^	^#^	^#^	^#^	^#^
MV		4	7.5	7.8	7.8	7.3	79.6
UV		3	6	6.7	5	3	54.4
	P-value		0.10	0.36	0.15	0.06	0.06
Asian		5	6.2	7.2	5	6	62.5
European		4	6.8	7.3	7.5	5*	69.7
	P-value		0.53	0.71	0.21	0.58	0.56

Abbreviation: NA, not assessed

^#^ p-value not calculated because of low study number

*included 2 studies without survival analysis

### Study results and meta-analysis

**Fig 2:**
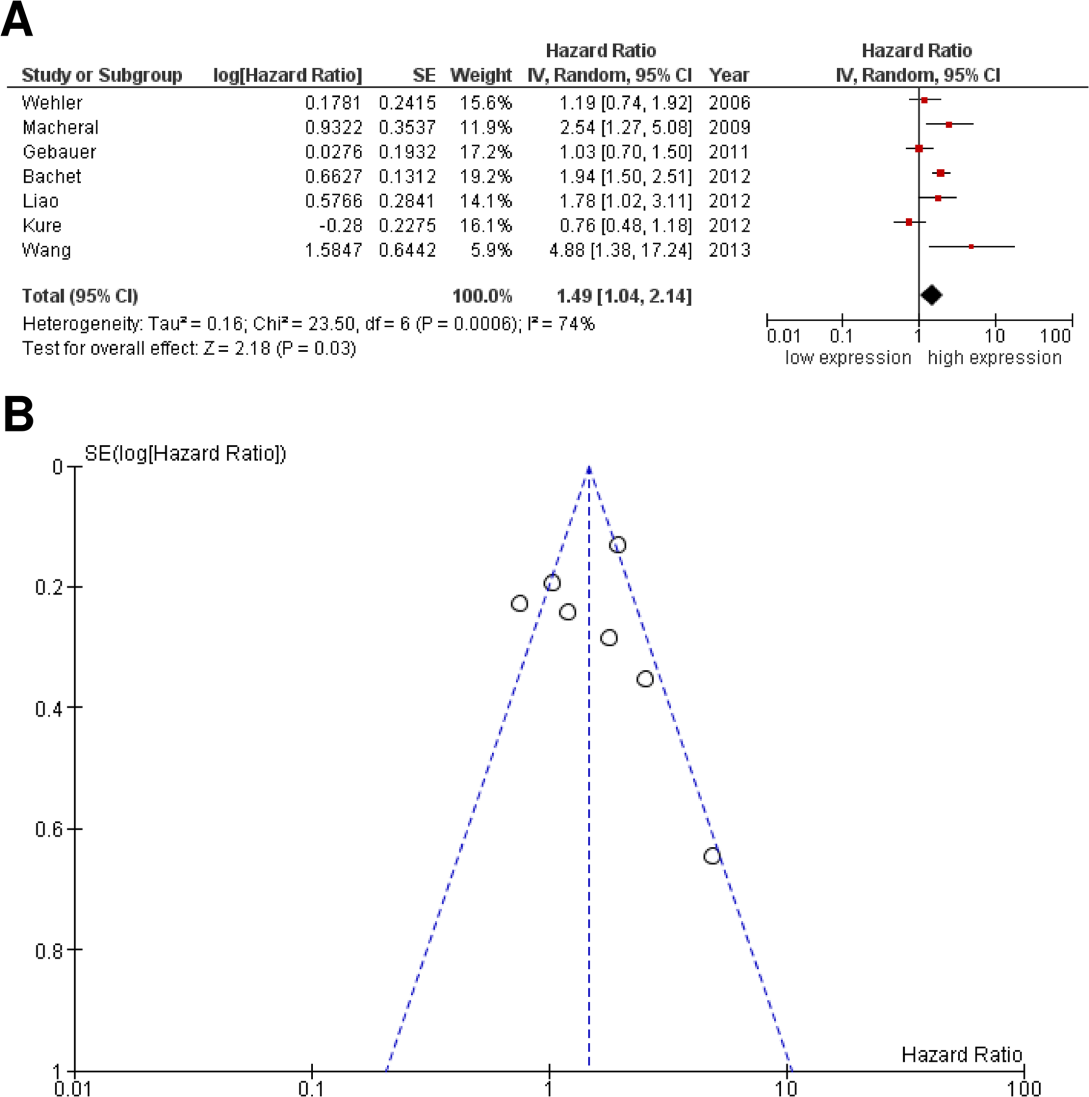
Meta-analysis comparing expression of CXCR4 with overall survival in patients with PDAC. **(A)** The Forest plot reflects the individual and pooled HR with CI. Heterogeneity was calculated by the Cochrane Q test (Chi-squared test; Chi^2^) and inconsistency (I^2^). **(B)** Funnel plot of included studies exhibited a symmetric distribution. Y-axis represents the standard error (SE), x-axis represents the study's result.

Initially, our aim was to analyze whether CXCR4 expression is a valid prognostic marker in patients with PDAC. Therefore, we pooled the extracted HRs from seven studies including 1101 patients with PDAC by using a random-effects model. As shown in **Fig 2A**, high CXCR4 expression was associated with poor overall survival (HR 1.49; 95% CI: 1.04–2.14; *P* = 0.03). However, Cochrane Q test (Chi^2^ = 23.50; *P* = 0.0006) as well as test of inconsistency (I^2^ = 74%) revealed a significant heterogeneity among the included studies. Visual inspection of the funnel plot did not reveal substantial asymmetry **(Fig 2B)**. To further assess whether a single study might be responsible for this heterogeneity, we simply excluded one study at a time and re-calculated the pooled HR (data not shown). However, one-way sensitivity analysis failed to identify an individual study that significantly reduced heterogeneity. Subsequently, we initiated subgroup analyses to reveal if heterogeneity was related to differences in HR estimation, quality of studies, and the country in which the studies were performed **(Table 3)**. Interestingly, subgroup analysis distinguishing between studies from Asian or European countries or sample size (≥ 103 versus < 103 patients) still reflected heterogeneity. Notably, studies of high quality using multivariate analysis confirmed the prognostic value of CXCR4 expression without reflecting heterogeneity. In contrast, studies estimating HRs by univariate analysis, reflected by a lower quality score, still failed to identify a significant context between CXCR4 and overall survival.

**Table 3:** Subgroup analyses evaluating methodological and demographic effects on the association between CXCR4 and overall survival in patients with PDAC.

					Pooled Data (Random)			Test for Heterogeneity Subgroup	
		No. of Studies	Cases	HR	95% CI	P-value	Chi^2^	P-value	I^2^ (%)
Cases (N)									
	≥ 103	4	885	1.18	0.76–1.92	0.46	16.38	0.0009	82
	< 103	3	216	2.28	1.46–3.56	0.0003	2.24	0.33	11
Survival analysis									
	UV	3	457	0.97	0.76–1.25	0.84	2.04	0.36	2
	MV	4	644	2.02	1.63–2.51	<0.00001	2.59	0.46	0
Global Quality Score									
	≥ 68.4	4	644	2.02	1.63–2.51	< 0.00001	2.59	0.46	0
	< 68.4	3	457	0.97	0.76–1.25	0.84	2.04	0.36	2
Country									
	Asian	3	250	1.61	0.66–3.92	0.30	10.78	0.005	81
	European	4	851	1.52	1.03–2.25	0.04	10.53	0.01	72

**Fig 3:**
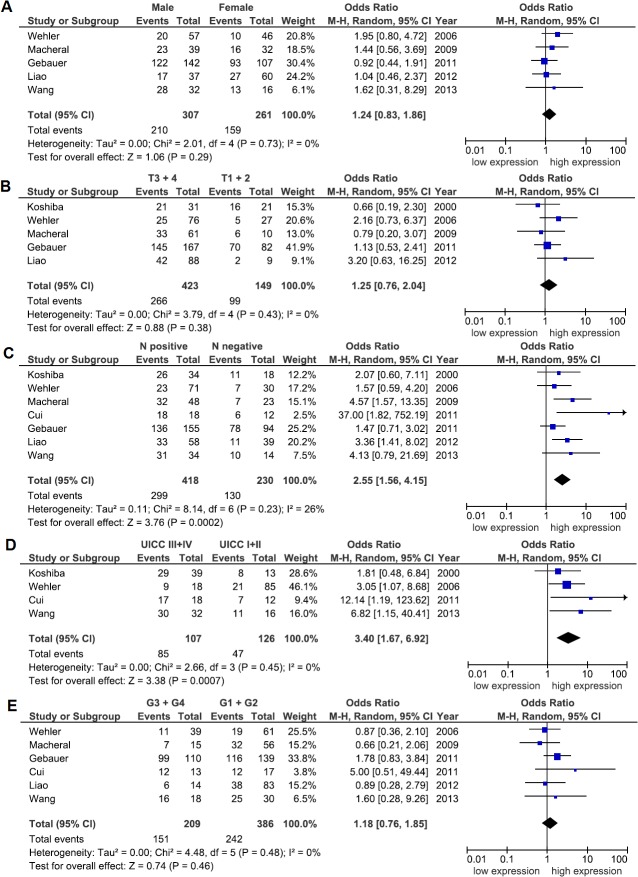
Association between CXCR4 and clinicopathological variables. The Forest plot reflects the individual and pooled OR with CI for the relationship between expression of CXCR4 and **(A)** sex, **(B)** depth of invasion (T-stage), **(C)** lymph node metastasis (N-status), **(D)** UICC tumor stage or **(E)** grade of differentiation. Heterogeneity was verified by the Cochrane Q test (Chi-squared test; Chi^2^) and inconsistency (I^2^).

**Fig 4:**
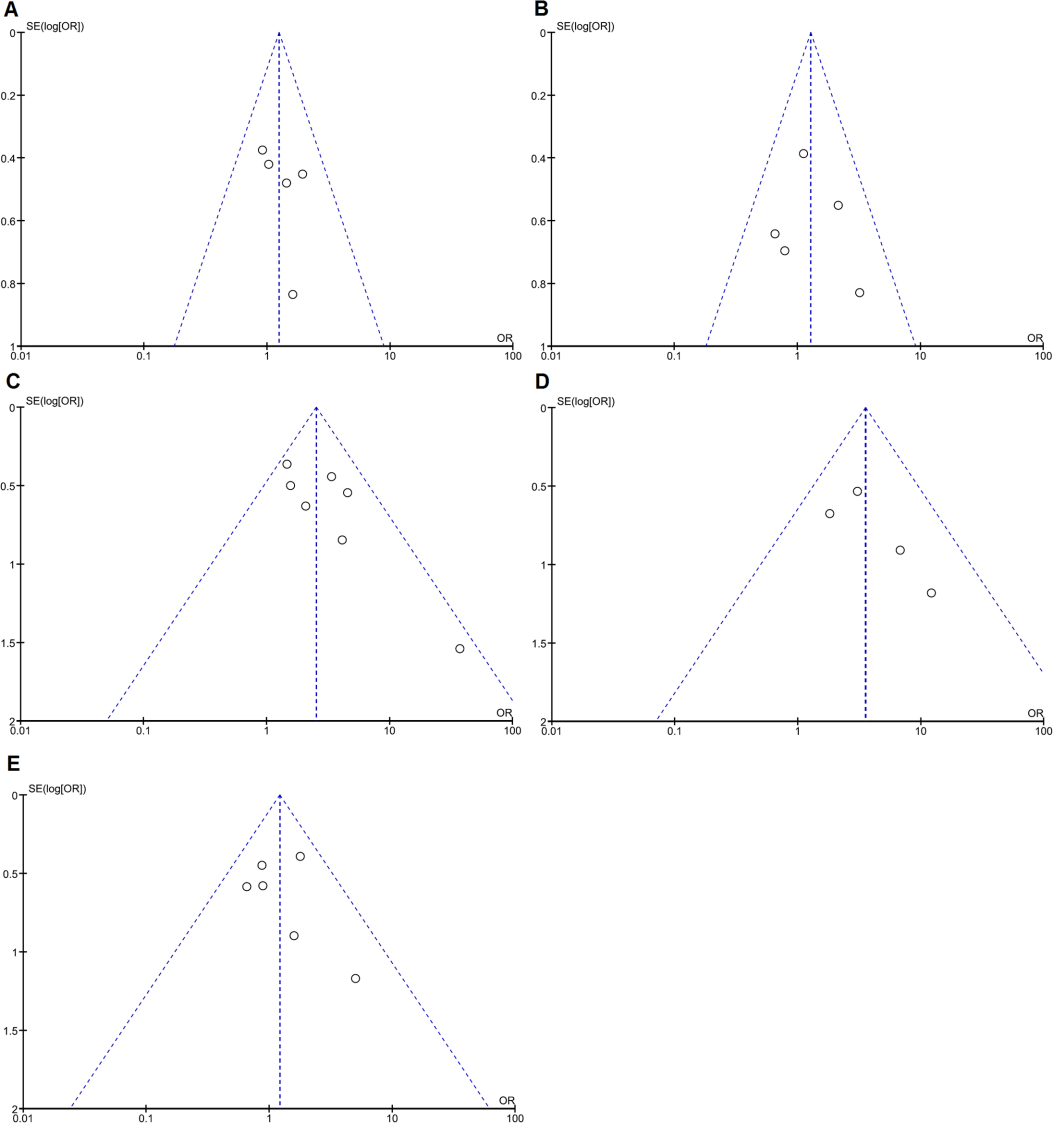
Analysis for potential publication bias. Funnel plots were prepared to assess publication bias with respect to the analyzed variable **(A)** sex, **(B)** depth of invasion (T-stage), **(C)** lymph node metastasis (N-status), **(D)** UICC tumor stage and **(E)** grade of differentiation. Y-axis represents the standard error (SE), x-axis represents the study's result.

**Fig 5:**
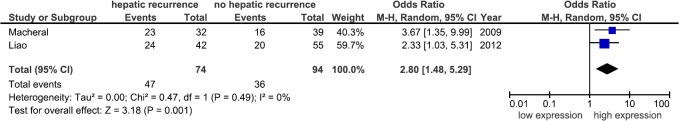
Association between CXCR4 and hepatic metastasis during follow up. Forest plot reflects the individual and pooled OR with CI to assess the association between CXCR4 and hepatic metastasis during follow up. Heterogeneity was quantified by the Cochrane Q test (Chi-squared test; Chi^2^) and inconsistency (I^2^).

Next, our goal was to elucidate a possible link between CXCR4 expression and clinicopathological parameters such as patients’ sex, UICC stage, depth of invasion, lymph node or distant metastasis as well as grade of differentiation in PDAC **(Fig 3)**. Again, a random-effects model was chosen to quantify the pooled ORs and CIs for each clinicopathological subgroup. Thus, CXCR4 expression positively correlated with lymph node metastasis (OR: 2.55; 95% CI: 1.56–4.15; *P* = 0.0002; I^2^ = 26%) and advanced UICC stage III and IV (OR: 3.40; 95% CI: 1.67–6.92; *P* = 0.0007; I^2^ = 0%) **(Fig 3C and 3D)**. Moreover, funnel plots did not reflect a substantial asymmetry **(Fig 4)**. From the six studies that correlated CXCR4 expression with distant metastasis, three studies obviously included only patients with metastasis to the liver [16, 17, 38]. Out of these studies, two studies defined hepatic metastasis as recurrence during follow up [16, 17]. In contrast, Bachet and colleagues focused on the association between expression of CXCR4 and distant recurrence [36]. The remaining two studies included distant metastasis without giving further information [6, 41]. When pooling the data from the two studies that clearly defined hepatic metastasis as recurrence during follow-up, we found an association between high CXCR4 expression and hepatic metastasis (OR: 2.80; 95% CI: 1.48–5.29; *P* = 0.001; I^2^ = 0%) **(Fig 5)**. Because of the small number of studies, an estimation of publication bias was not performed.

## Discussion

Increasing evidence indicates that chemokines not only induce antitumor actions by stimulating the immune system but also activate tumor cells by directly influencing tumor transformation, survival, and proliferation as well as migration, invasion, and metastasis [42]. In this context, the CXCR4/CXCL12 interaction has been demonstrated to direct CXCR4+ tumor cells to the sites for the development of metastasis [43]. Under hypoxic conditions within the tumor mass, hypoxia-inducible factor-1a (HIF-1a) becomes up-regulated, which in turn induces high expression of CXCR4 on tumor cells [44]. Consequently, this observation implies that CXCR4-expressing tumor cells are directed along a cytokine gradient to CXCL12-expressing organs to form metastasis [45]. In addition to chemotaxis, binding of CXCL12 to CXCR4 induces distinct signalling pathways and initiates a cascade of cellular processes related to proliferation and survival, increases the intracellular calcium level, and also interferes in the regulation of gene transcription [46]. So far, numerous studies have demonstrated the expression of CXCR4 in different types of gastrointestinal neoplasia such as gastric cancer and colorectal cancer [7, 8]. Recently, meta-analyses strongly supported the prognostic and clinicopathological relevance of CXCR4 in breast, esophageal, colorectal, gastric, and ovarian cancer, as well as in renal cell carcinomas and gliomas [47–53]. However, in PDAC the published studies presented conflicting data when CXCR4 was analyzed for its relevance as prognostic biomarker. Thus, summarizing the currently available literature by meta-analysis was important to provide a better overview on the association of CXCR4 with patients overall survival and clinicopathological variables. Accordingly, we defined inclusion and exclusion criteria and performed an extensive literature search of studies that were eligible to be included into a meta-analysis that aimed to provide evidence on the role of CXCR4 as a biomarker in PDAC. Moreover, the objective of our meta-analysis was to rigorously evaluate the quality of the included studies and to assess potential sources of heterogeneity by performing subgroup analysis. Finally, we were able to include 1183 patients from nine eligible studies that were published between 2000 and 2013. Interestingly, our meta-analysis confirmed that the incidence of positive lymph nodes was accompanied by a high expression of CXCR4 in primary tumors. These data are in agreement with most of the recently published meta-analyses that demonstrated an association between CXCR4 expression and metastatic status in renal cell carcinoma, breast cancer, gastric cancer, colorectal cancer and esophageal cancer [47, 50–53]. More importantly, the observation that CXCR4 expression in ex vivo PDAC samples correlates with patients metastasized disease stage together with current research on experimental tumor models supports the involvement of CXCR4 in the formation of metastasis. Unfortunately, because the data from the included studies dealing with distant metastasis were heterogeneous (i.e. side of distant metastasis, time point of diagnosis) we were unable to robustly analyze the association of CXCR4 with distant metastasis. Hence, we were able to pool the data from only two studies that focused on the association of CXCR4 with hepatic metastasis as recurrence during follow up. Although these data might support an association of CXCR4 with the incidence of hepatic metastasis, due to the limited number of includable studies these data must be interpreted with caution.

Undoubtedly, when pooling the HR of all studies the association between high expression of CXCR4 and poor prognosis in patients with PDAC was accompanied by strong heterogeneity. However, as revealed by one-way sensitivity analysis, heterogeneity was not caused by any single study. Importantly, high-quality studies that performed multivariate analysis homogeneously confirmed our initial results that high expression of CXCR4 was associated with poor prognosis. Thus, the source of heterogeneity appears to be related to studies of lower quality that presented only univariate analysis by illustrating Kaplan-Meier survival curves.

Recently, by identifying another transmembrane receptor CXCR7 that binds CXCL12, CXCR4 actions have been under debate. In this context, it has been proposed that CXCR7 might modulate CXCR4 signaling by simply acting as a “decoy” for CXCL12. Thus by competing with CXCR4 for binding CXCL12, CXCR7 has been proposed to sequester CXCL12 which would impact the chemoattraction and migration of CXCR4 positive cells [54, 55]. On the other hand, others reported that CXCL12 signaling was regulated by heterodimerization of CXCR7 with CXCR4 [56]. In addition, Hernandez et al. reported that CXCR4 promoted invasion of tumor cells in a breast cancer model whereas CXCR7 impaired invasiveness. Moreover, they demonstrated that CXCR7 enhanced tumor growth by activating the process of angiogenesis. Interestingly, CXCR7 expressing breast cancer cells not only enhanced proliferation of CXCR4 positive breast cancer cells but also supported spontaneous metastasis [57]. However, out of the eligible studies that could be included in this meta-analysis only Cui and colleagues investigated the expression of both CXCR4 and CXCL12 in PDAC [37]. In contrast, Marechal [17] and Gebauer [6] performed IHC for both CXCR4 and CXCR7 in their cohort of PDAC patients. In this context, Gebauer did not find any significant association between a positive staining for CXCR7 alone or together with CXCR4 and overall or disease free survival. In contrast, Marechal reported that high co-expression of CXCR4 and CXCR7 was associated with poor prognosis. Consequently, because of the low number of studies that analyzed the co-expression of CXCR4, CXCR7 and CXCL12 in PDAC, we were unable to perform a meta-analysis relative to the regulation of the CXCR4 pathway by CXCR7 or CXCL12 expression.

However, this meta-analysis has some limitations. First, because survival data were extracted from Kaplan-Meier curves we might have introduced a bias, which resulted in the pronounced heterogeneity among the studies. On the one hand, this is explainable by the fact that HRs calculated from extracted data are definitely less accurate than reported HRs from multivariate analysis. In addition, for the extracted survival data we assumed that the number of censored cases was constant during the period of follow-up. On the other hand, none of the three studies that presented results from univariate analysis found a correlation between overall survival and CXCR4. Second, further sources of a relevant bias that might significantly affect the results of this meta-analysis might be explainable by the retrospective study design of all included studies and by including only articles written in English, which are more likely to be published when reporting positive results in contrast to studies with negative results. Thus, non-English written articles that might be potentially eligible were not included. Third, detection and quantification of CXCR4 was not performed by a validated and standardized method. This is reflected by the heterogeneous definition of the cut of values used in the different studies which might be not completely accounted by the use of the random effects model. Fourth, the power of the funnel plots in estimating publication bias might be influenced by the limited number of eligible studies for meta-analysis. Since we included less than 10 studies our funnel plots might not clearly distinguish chance from real asymmetry and therefore have to be interpreted with caution.

However, our meta-analysis supports the ongoing research that identified CXCR4-initiated signalling pathways as potentially druggable targets in cancer therapies. In this context, the bicyclam AMD3100 has been shown to specifically antagonize CXCR4 by inhibiting CXCL12 binding. Thus, AMD3100 attenuated successfully CXCL12-induced chemotaxis, cell motility, and proliferation, as well as resistance to gemcitabine in pancreatic cancer cell lines. In the future, clinical trials must still show the benefit of a targeted therapy against components that are involved in CXCR4 signalling in PDAC.

In conclusion, by performing a comprehensive literature search and meta-analysis we could demonstrate that CXCR4 expression levels were related to metastatic disease and overall survival in patients with PDAC. In particular, high-quality studies presenting data from multivariate analysis provided evidence for the prognostic value of CXCR4 in PDAC. Nonetheless, in the future large multicenter and prospective trials of high quality using a validated detection method and that evaluate also CXCR7 as well as CXCL12 are needed to further confirm the role of CXCR4 as biomarker in PDAC disease.

## Supporting Information

S1 PRISMA Checklist (DOC)
